# People With Cancer Experience Worse Psychosocial and Financial Consequences of COVID-19 Compared With Other Chronic Disease Populations: Findings From the International COVID-19 Awareness and Response Evaluation Survey Study

**DOI:** 10.1200/GO.23.00085

**Published:** 2023-12-07

**Authors:** Mohamad Baydoun, Andrew I.G. McLennan, Linda E. Carlson

**Affiliations:** ^1^ Faculty of Nursing, University of Regina, Regina, SK, Canada; ^2^ Department of Psychology, University of Regina, Regina, SK, Canada; ^3^ Department of Psychosocial Oncology, Cumming School of Medicine, University of Calgary, Calgary, AB, Canada

## Abstract

**PURPOSE:**

The COVID-19 pandemic is likely to have profound psychosocial impacts across the globe. In this analysis of the International COVID-19 Awareness and Response Evaluation (iCARE) survey study, we comparatively investigated the psychosocial effects of COVID-19 on individuals with cancer and people with other chronic illness.

**METHODS:**

iCARE study respondents were divided into two groups on the basis of self-reported health status: (1) active/current cancer (with or without any other chronic condition: heart disease, lung disease, hypertension, diabetes, severe obesity, immunity disease, and depressive or anxiety disorder); and (2) other chronic condition, but not cancer. Linear regressions were conducted to evaluate the associations between health status and outcomes.

**RESULTS:**

Worldwide, 18,154 iCARE study respondents (mean age, 50.8 years) from 175 countries were included in the analysis. Among them, 3.8% (n = 677) identified as having active/current cancer and 96.2% (n = 17,477) identified as having other chronic condition. Multivariate analyses showed significant associations between having cancer and declined mental (β = .364; *P* < .0001) and physical (β = .317; *P* < .0001) health since the start of the COVID-19 pandemic, relative to those with other chronic illness. Moreover, individuals with cancer demonstrated a higher likelihood of reporting maladaptive coping mechanisms such as increased alcohol use (β = .457; *P* < .0001) and financial hardships such as not paying rent/mortgage (β = .476; *P* < .0001), compared with people with other chronic illness.

**CONCLUSION:**

Individuals with cancer worldwide tended to have worse psychosocial and financial challenges during the COVID-19 pandemic, compared with other chronic disease populations. Clinicians need to be aware of the importance of attending to the specific mental health needs of individuals with cancer during and after COVID-19–related restrictions.

## Teaser

Our study aims to understand how psychological and behavioral responses to the COVID-19 pandemic differ between people with cancer and those with other chronic illnesses across 175 countries. We recommend clinicians place an emphasis on addressing mental health gaps.

## INTRODUCTION

Across the general population and all ages, COVID-19 has given rise to high psychological distress and deteriorated mental health.^1,2^ Numerous general population studies have showed significant levels of fear and anxiety surrounding health outcomes for oneself and others, unemployment, and financials issues, as well as major adjustments to lifestyle (social distancing, persistent lockdowns, etc).^1,2^

**CONTEXT**

**Key Objective**
Our study aims to address the following question: “How do the psychological and behavioral responses to the COVID-19 pandemic vary between patients with cancer and those with other chronic illnesses globally?” Unlike previous, smaller-scale studies, our multinational analysis of the psychosocial experiences of these groups gives stakeholders valuable insights for tailored interventions.
**Knowledge Generated**
Globally, during the COVID-19 pandemic, individuals with cancer faced more psychosocial distress and disruptions in medical care than those with other chronic illnesses. Nonetheless, individuals with cancer reported a mix of coping behaviors, adopting both negative mechanisms, such as increased use of recreational drugs, and positive strategies, including a healthier diet and more exercise, suggesting a nuanced and complex response to pandemic-induced challenges.
**Relevance**
Clinicians should prioritize addressing gaps in mental health and psychosocial support for individuals with cancer and seek strategies to promote healthier lifestyles and resilience in the face of future similar global events.

For those suffering from chronic illness such as cancer, diabetes, and cardiovascular disease, the psychosocial impacts may be more significant than for the general population. Furthermore, individuals with chronic illness might also have experienced increased psychological distress while managing their conditions amid the pandemic.^3-6^

For individuals living with cancer, the adverse effects of COVID-19 on mental health may be even more severe.^5^ A recent multinational general population survey study (N = 41,212) showed that relative to healthy individuals, significant associations have been found between having cancer and symptoms of anxiety and depression, as well as worsened overall quality of life (QOL) during the COVID-19 pandemic.^7^ Patients receiving active cancer treatment are often immunocompromised, potentially making them vulnerable to COVID-19 disease or related health complications.^4^ In addition, many individuals with cancer are required to visit health centers frequently, sometimes on a daily basis, thereby increasing their risk of exposure to the virus. Caregiver access may also be reduced in many circumstances for both inpatients and outpatients, thereby impeding the vital support that patients often require. Moreover, patients may be fearful of their own disease prognosis and status as many appointments deemed nonessential have been delayed since the start of the pandemic.

For those living with chronic conditions and cancer, increases in unhealthy behavior can have more serious implications for disease prognosis, recovery, and general well-being.^8-10^ Recent studies show that health behaviors (eg, substance use and smoking) have been adversely affected by COVID-19.^2,11^ Forced changes to lifestyle, including less social contact, alongside the repercussions of untreated COVID-19–related psychological comorbidity, may contribute to uptake of unhealthy coping behaviors (eg, alcohol abuse).^8-12^

Over the past 3 years, the impacts of the COVID-19 pandemic have left an indelible mark on mental health among people with chronic illness and those with cancer specifically.^8-10^ Individuals with cancer may face a unique set of stressful circumstances that other chronic disease patients may not experience. To our knowledge, to date, no comparative studies have evaluated the psychosocial impact of COVID-19 on individuals with cancer and chronic disease populations on a global scale. Sample sizes of previous studies have also generally been quite small.^8-12^ This study benefits the literature by providing novel data regarding the comparative psychosocial well-being and subsequent health behaviors between these two groups in a large, multinational survey sample. This study can aid in understanding what type of psychosocial support is needed, and for which individuals, and provide further insight to the physical, psychological, and social health outcomes of the COVID-19 pandemic.

### Objectives

The objectives of this analysis of the International COVID-19 Awareness and Response Evaluation (iCARE) survey data^13^ are to

1. Compare the impacts of COVID-19 on psychosocial health and QOL between individuals with cancer and people with other chronic illness across diverse countries of the globe.
2. Compare the health behaviors (eg, substance use and physical activity) during the COVID-19 pandemic between individuals with cancer and people with other chronic illness across diverse countries of the globe.

## METHODS

### iCARE Study

The iCARE study^14^ is an ongoing study led by investigators from the Montreal Behavioural Medicine Centre in collaboration with over 200 researchers from more than 40 countries.^13^ The study uses a cross-sectional, multicohort survey design and primarily aims to capture key data on people's awareness, attitudes, and behaviors as they relate to COVID-19 policies.^13^

### Study Design

**FIG 1:**
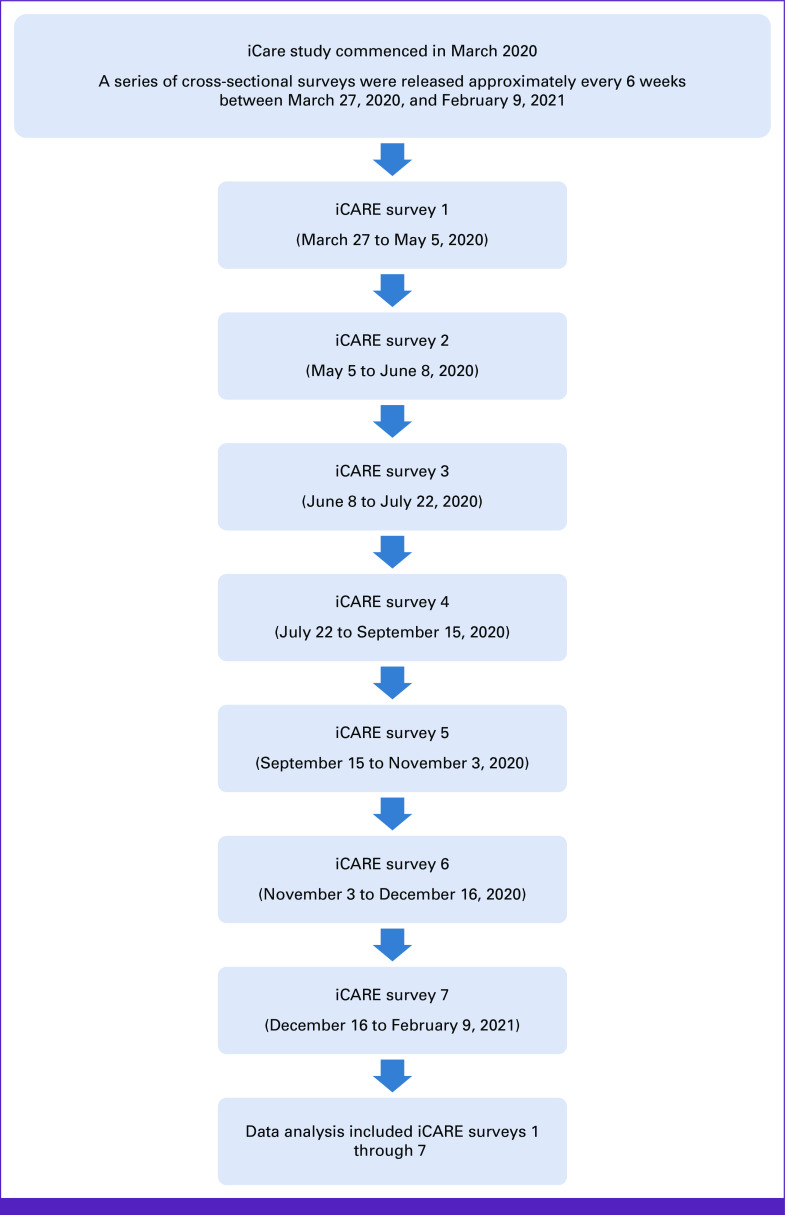
Study flow diagram. iCARE, International COVID-19 Awareness and Response Evaluation.

Using a cross-sectional, multicohort design, the current analysis covered iCARE surveys 1 through 7 and comprised data collected at multiple time points, spanning from March 27, 2020 (survey 1 of iCARE), to February 9, 2021 (survey 7 of iCARE; Fig 1).

### Participants

This analysis was carried out on data collected from a convenience sample of adult participants (age 18 years and older) from 175 countries. The data set included 18,154 adults (age 18 years and older) across the globe who responded to the iCARE survey between March 27, 2020, and February 9, 2021.

### Study Instrument

Because of the unavailability of validated scales evaluating outcomes in relation to COVID-19 when the iCARE study was launched, an iCARE survey was developed specifically by the iCARE investigators. The survey (Data Supplement, File S1) was designed to measure constructs consistent with the COM-B model, a health psychology theory that cites capability (C), opportunity (O), and motivation (M) as three key factors leading to behavior (B) change,^15^ and the health belief model, a psychological and behavioral theory that posits six constructs to predict health behavior: risk susceptibility, risk severity, benefits to action, barriers to action, self-efficacy, and cues to action.^16^

### Data Collection

Since its launch on March 27, 2020, 12 variations of the iCARE survey have been distributed in waves approximately 6 weeks apart.^15^ Survey data are collected by all global collaborators using a convenience sampling approach (globally) and parallel representative sampling in countries where funds are available.^15^ The survey is available in 34 languages and distributed through a number of channels, including professional organizations and networks, social media platforms, universities and schools, health care settings, and community organizations.^13^

### Variables

To address our research objectives, the following variables from the iCARE survey data were included in the analysis.

Sociodemographic information: Sociodemographic characteristics collected from the iCARE survey data included sex, age, type of residential area (urban/rural/suburban), level of education, and employment status.

Health status: To analyze the stated research objectives, we used information on respondents' health status to differentiate between individuals with cancer and other chronic illness patients. Specifically, the iCARE survey included a question that asked respondents to report any chronic mental or physical health condition, including cancer, heart disease, lung disease, hypertension, diabetes, severe obesity, immunity disease, and depressive or anxiety disorder. On the basis of self-reported health status, we divided respondents into two groups: (1) active/current cancer with or without any other chronic mental or physical health condition and (2) no active/current cancer but has one or more other chronic mental or physical health condition.

Psychosocial health, QOL, and health behaviors: For the purposes of comparing the psychosocial implications of COVID-19 between individuals with cancer and other chronic disease populations, we used the following questions from the iCARE survey data to evaluate participants' psychosocial health, QOL, and health behaviors during the pandemic.

#### Objective 1: Impacts of COVID-19 on Psychosocial Health and QOL

1. Question 1: 19 items assessing COVID-19–related concerns (Because of COVID-19, I am concerned about being infected myself; the impact of being infected on my health, including dying, etc), each with four answer options (To a great extent; Somewhat; Very little; Not at all).
2. Question 2: 19 items assessing the psychosocial consequences of COVID-19 (Because of COVID-19, I have felt sad, depressed, or hopeless; etc), each with four answer options (To a great extent; Somewhat; Very little; Not at all).
3. Question 3: one item assessing QOL (How has your overall QOL changed as a result of the COVID-19 pandemic?) with five answer options (It's gotten much better; It's gotten better; It's remained the same; It's gotten worse; It's gotten much worse).

#### Objective 2: Impacts of COVID-19 on Health Behaviors

1. Question 4: six items assessing health behaviors (How have the following behaviors changed since the start of COVID-19: Doing physical activity; Eating a healthy diet; etc), each with five answer options (I do this a lot more; I do this more; I do this as much as before; I do this less; I do this a lot less).

### Data Analysis

Data were analyzed using SAS statistical software package (version 9.4, SAS Institute Inc., Cary, NC). The means and standard deviations from each group (cancer and chronic illness) were first summarized using descriptive statistics. Means/analysis of variance for continuous data and frequencies/chi square tests for categorical data were used to compare sociodemographic characteristics between groups. Generalized linear model procedure and least-squares means for multiple comparisons (LSMEANS with Bonferroni correction) were applied to evaluate the association of health status (cancer and other chronic illness) with psychosocial health, QOL (objective 1), and health behavior (objective 2) outcomes. Sociodemographic variables were included in the models as covariates. To mitigate the impact of potential confounding factors, a propensity-matched analysis was applied between the two groups: active/current cancer and other chronic conditions. The variables used in the propensity score model included age, sex, employment status, and educational level. The propensity score matching was conducted using the multinomial propensity scores (mnps) function within the R statistical software package (R Foundation, Vienna, Austria). Imputation of missing values was performed using predictive mean matching.

### Ethical Considerations

The iCARE study was approved by the Research Ethics Committee at the Centre intégré universitaire de santé et de services sociaux du Nord-de-l’Île-de-Montréal (CIUSSS-NIM; REB No.: 2020-2099/03-25-2020). The current analysis was approved by the Health Research Ethics Board of Alberta (HREBA)—Cancer Committee (CC; REB No.: HREBA.CC-21-0267). The iCARE survey did not include any potentially identifiable information, such as names or contact details. Subject consent was waived because of the nonidentifiable nature of the collected data. The present paper is presented in line with the Consensus-Based Checklist for Reporting of Survey Studies^17^ (Data Supplement, File S2).

## RESULTS

### Participants

Worldwide, 18,154 iCARE study respondents (mean age, 50.8 years; 28% male and 72% female) from 175 countries were included in the analysis. Among them, 3.8% (n = 677) identified as having active/current cancer and 96.2% (n = 17,477) identified as having other chronic condition. There were significantly more females than males in both groups (*P* < .001). The majority of respondents across both groups reported living in an urban area (*P* < .001) and attending college or university compared with only high school (*P* < .001). Respondents with active/current cancer were significantly older (mean age, 54.5 years) than those identified as having other chronic illness (mean age, 47.1 years; *P* < .001). Additionally, the majority of individuals with cancer were retired or homemakers (48.5%), whereas most of the respondents with other chronic illness were employed (37%; *P* < .001). Participant characteristics are summarized in Table 1.

**TABLE 1:**
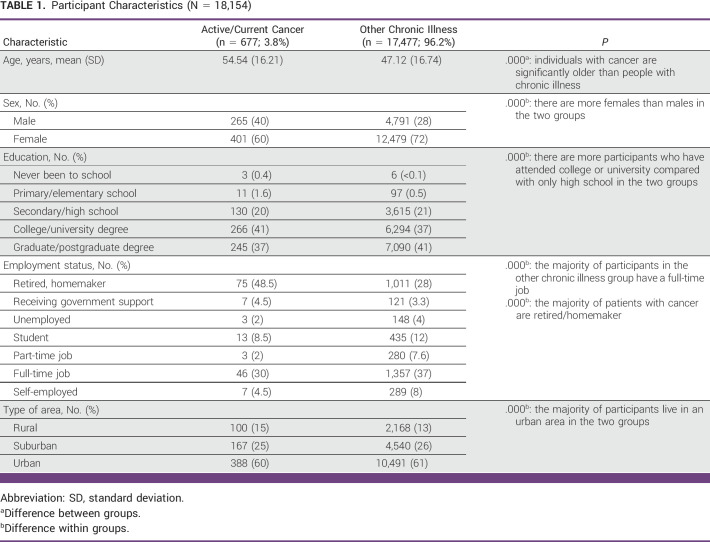
Participant Characteristics (N = 18,154)

### Objective 1: Impacts of COVID-19 on Psychosocial Health and QOL

Table 2 shows the results of the multivariate analyses, examining the associations between health status and psychosocial outcomes. Individuals with cancer were more likely than other chronic disease populations to report arguments with family members (β = .243; *P* < .0001), suspicion and distrust of others (β = .182; *P* < .0001), getting separated or divorced (β = .645; *P* = .004), declined mental health (β = .364; *P* < .0001), and declined physical health (β = .317; *P* < .0001) since the start of the COVID-19 pandemic. Similarly, relative to people with other chronic conditions, people with active or current cancer were more likely to report concerns regarding contracting COVID-19 (β = .140; *P* < .0001), dying from COVID-19 (β = .144; *P* < .0001), and not being able to see family or friends due to COVID-19 restrictions (β = .209; *P* < .0001). In addition, individuals with cancer more often reported interruptions in non–COVID-19–related medical care, including having medical appointments canceled (β = .107; *P* < .0001) and trouble receiving medical care (β = .214; *P* < .0001), relative to people with other chronic illness.

**TABLE 2:**
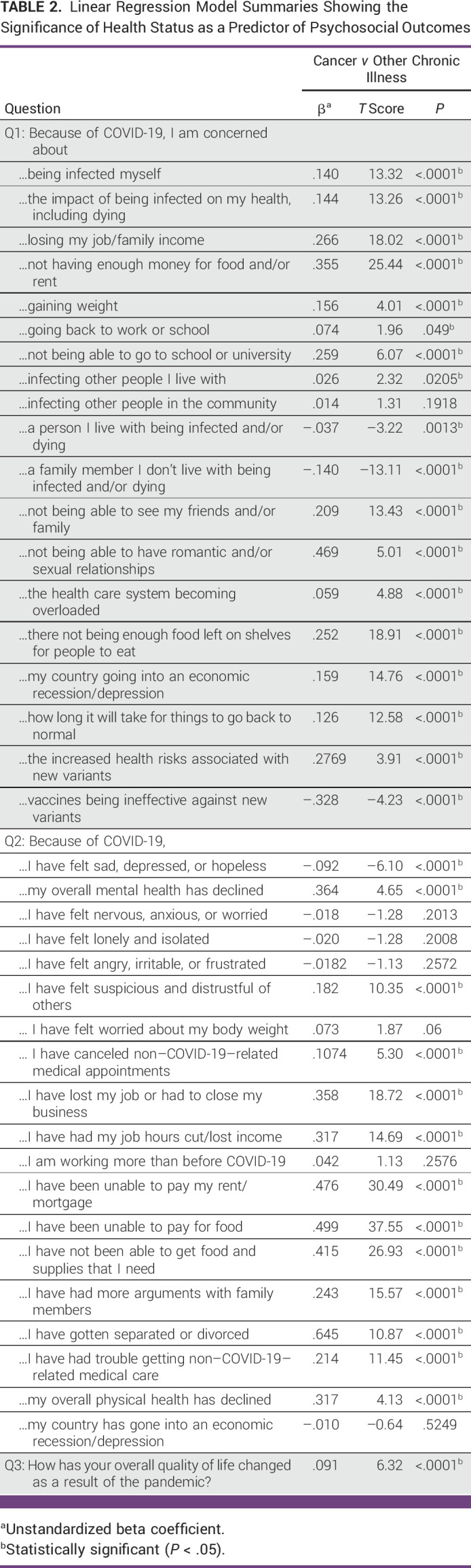
Linear Regression Model Summaries Showing the Significance of Health Status as a Predictor of Psychosocial Outcomes

Furthermore, in comparison with other chronic disease populations, individuals with cancer were more likely to report concerns regarding their ability to go to school or university (β = .074; *P* < .05), finding enough food left on shelves (β = .252; *P* < .0001), and the time needed for life to return to a prepandemic normal (β = .126; *P* < .0001). Similarly, individuals with cancer were more likely to experience financial hardships than those living with other chronic illness, including inability to pay rent/mortgage (β = .476; *P* < .0001) and inability to pay for food/supplies (β = .499; *P* < .0001). Nonetheless, when asked about changes in overall QOL due to COVID-19, respondents with chronic illness were more likely than those active/current cancer to choose a negative response (It's gotten worse; It's gotten much worse; β = .091; *P* ≤ .0001).

### Objective 2: Impacts of COVID-19 on Health Behaviors

The results of the multivariate analyses evaluating the relationships between health status and health behaviors are shown in Table 3. Respondents with active/current cancer were more likely than those with other chronic illness to report negative coping approaches, such as increased alcohol use (β = .45; *P* < .0001) and increased cigarette smoking (β = .54; *P* < .0001) since the start of the COVID-19 pandemic. We also found a significant association between having active or current cancer and increased utilization of positive coping mechanisms (ie, doing physical activity and eating healthy diet) during the COVID-19 pandemic (*P* > .0001).

**TABLE 3:**
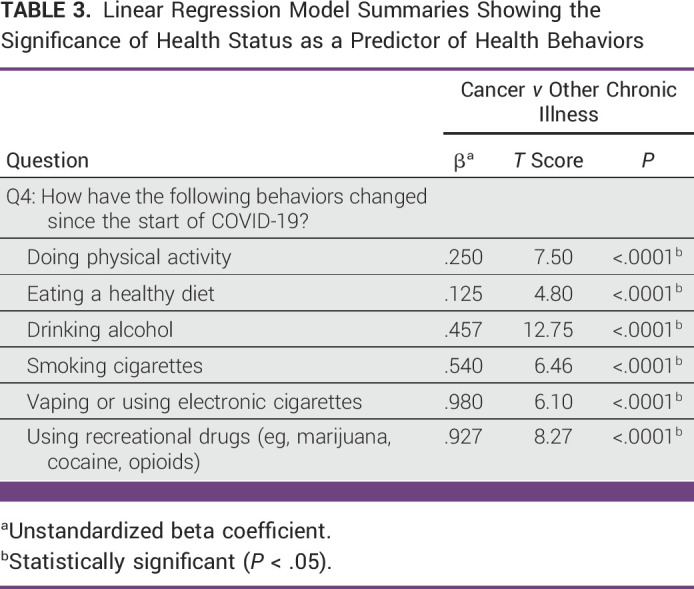
Linear Regression Model Summaries Showing the Significance of Health Status as a Predictor of Health Behaviors

## DISCUSSION

The purpose of this study was to comparatively investigate the impacts of COVID-19 on adults living with cancer and adults living with chronic illness, as well as to compare the health behaviors of these two groups during the COVID-19 pandemic. The results demonstrate that an active diagnosis of cancer was significantly associated with adverse COVID-19–related psychosocial symptoms. On a global scale, those with cancer experience further decline in mental health than those with other chronic conditions. This is not atypical for this population when compared with other groups, as the prevalence of comorbid psychosocial conditions are higher among people living with cancer generally.^18,19^

In a recent systematic review of COVID-19–related anxiety prevalence in individuals with cancer, the authors found that rates of psychological distress and comorbid disorders have increased during the pandemic and were related to fears of worsened conditions, delays and interruptions in treatment schedules, and fear of contracting COVID-19.^20^ Findings from our much larger international study align with this review, as those with cancer experienced more psychosocial issues related to COVID-19, such as fear of dying from the virus, concerns about interrupted medical care, and experiencing separation or divorce.

The QOL for many individuals with cancer might have been affected by the COVID-19 pandemic, primarily because of increased levels of isolation and loneliness.^21,22^ Results of an American survey study of 187 individuals with cancer found that 53% were reportedly lonely on the University of California, Los Angeles, loneliness scale.^23^ Findings from the current study corroborate that, relative to other chronic disease populations, individuals with cancer were more prone to reporting difficulties in seeing family or friends due to COVID-19–related restrictions. However, our results show that individuals with other chronic illness have experienced a more significant decrease in their QOL since the start of the COVID-19 pandemic, compared with those with cancer. One possible explanation for this finding, which contradicts the majority of our results regarding psychosocial aspects, could be that individuals with cancer might have already been experiencing a compromised QOL before the onset of COVID-19. As a result, the significant impacts of the pandemic on their well-being might not have been perceived as dramatically altering their preexisting poor QOL.

In addition to higher rates of psychosocial problems, financial hardship was found to be of particular concern, where an inability to pay rent/mortgage, troubles paying for food/supplies, and general fears of not having enough money for food or rent were reported significantly more by people living with cancer. Financial hardship is a common issue experienced by many older adults who are no longer in the workforce as well as by others who are unemployed; however, the prevalence of financial hardship is indisputably more common for people living with cancer, as is supported in our findings where individuals with cancer were significantly older than the other group and nearly half (48.5%) were retired or homemakers. In addition to a lack of consistent income, individuals with cancer are often burdened with expensive treatments, medications, and inpatient costs, which for many can be financially crippling.^24^

The negative psychosocial and financial outcomes observed among individuals with cancer may partially explain our findings that utilization of certain negative health behaviors (eg, increased alcohol use) was significantly associated with having cancer, compared with people with other chronic illness. Nevertheless, people with cancer also reported an increased adoption of positive coping strategies, such as maintaining a healthy diet and engaging in physical activity. These mixed results might be attributed to the various coping approaches among participants in the initial year of the pandemic, alternating between positive and negative strategies.

There are several noteworthy limitations of this study. First, individuals reporting any chronic illness (excluding an active cancer diagnosis) were broadly categorized into one group. Furthermore, since the iCARE study was not designed to specifically assess the impact of the pandemic on individuals with cancer, the single question about cancer was general (active/current cancer), and in addition to not providing nuance into the type or stage of disease, the survey might not have captured all those with a history of a past cancer diagnosis. Hence, the results are largely applicable to current patients, rather than cancer survivors more generally. More interesting findings may have been discovered concerning relationships between various cancer types or specific chronic illnesses (eg, diabetes and heart disease) and the impacts of COVID-19 if data were available. Third, because it was not possible to know the number of people who received the study invitation, the response rate could not be measured. Fourth, response bias might have influenced the results. Last, although not evaluating the psychometric properties of the study survey might be considered a limitation, the iCARE study investigators made this choice to capture relevant constructs in real time.

Notwithstanding the above limitations, this study provides unique insight to the psychological, social, and financial challenges that individuals with cancer have faced since the start of the COVID-19 pandemic. Comparing these data to adults with other chronic illness has enabled a point of reference to be made, which, as discerned from our findings, indicates the need for better resources, assistance, and overall awareness for individuals with cancer during the pandemic. Our study provides evidence that necessary steps need to be taken to identify those living with cancer who are facing considerable hardship, and furthermore, provide the much-needed health and social services. However, we recognize that in some countries, limitations in the social safety net might hinder the ability to carry out these actions effectively. There are several important future steps for this research. First, a further investigation of the gaps in provision of mental health services in the cancer system and the avenues to make these services more accessible to patients is warranted. Moreover, scientific inquiry on strategies to promote and maintain healthy lifestyles for individuals with cancer during the pandemic would dually serve to benefit this vulnerable patient population now and in the case of future similar global events.

In conclusion, the current analysis of the iCARE study found that people with cancer were significantly older and more likely to be retired or unemployed, compared with other chronic disease populations. Relative to people with other chronic illness, those with cancer were found to have more adverse psychological symptoms related to COVID-19, as well as more social problems such as separation, divorce, and increased distrust of others. Additionally, those with cancer were more susceptible to financial hardship. Oncology and primary care clinicians need to be aware of the importance of attending to the psychosocial challenges experienced by individuals with cancer during the future restrictions related to this or future pandemics.
